# Determinants of the continuum of maternal healthcare service utilization in Nigeria: Evidence from the 2024 Nigeria Demographic and Health Survey

**DOI:** 10.1371/journal.pone.0359946

**Published:** 2026-10-05

**Authors:** Gideon Ikemdinachi Nwankwo, Rejoice Uche Obiora, Delight Mawufemor Agbi, Bisola Olubiyi, Daniel Mumba, Herbert Tafadzwa Kazonda, Isaac Isiko, Edson Mwebesa, Charles Natuhamya

**Affiliations:** 1 Department of Health Management and Policy, Faculty of Health Sciences, American University of Beirut, Beirut, Lebanon; 2 College of Health, Lehigh University, Bethlehem, Pennsylvania, United States of America; 3 Department of Mathematics, Physics and Computing, School of Science and Aerospace Studies, Moi University, Eldoret, Kenya; 4 Usher Institute, University of Edinburgh, Edinburgh, United Kingdom; 5 University of Global Health Equity, Kigali, Rwanda; 6 Malawi Liverpool Wellcome Programme, Infection and Immunity Research Group, Blantyre, Malawi; 7 Department of Economics and Finance, Great Zimbabwe University, Masvingo, Zimbabwe; 8 Faculty of Medicine, School of Population and Public Health, University of British Columbia, Vancouver, Canada; 9 Department of Mathematics, Faculty of Science, Muni University, Arua, Uganda; 10 Uganda Youth Development Link, Kampala, Uganda; Universidad Peruana Cayetano Heredia Facultad de Medicina, PERU

## Abstract

**Background:**

Maternal mortality remains a major public health challenge, particularly in low- and middle-income countries, with Sub-Saharan Africa bearing a disproportionate burden. Nigeria accounts for over one-quarter of all maternal deaths worldwide. This reflects gaps in access to and continuity of maternal healthcare. The continuum of maternal healthcare, which includes antenatal care (ANC), skilled delivery, and postnatal care (PNC) is critical for improving maternal and neonatal outcomes. This study examines the determinants of continuum of maternal healthcare service utilization in Nigeria.

**Methods:**

This study utilized data from 13,596 women (weighted n = 14,183) from the 2024 Nigeria Demographic and Health Survey. Maternal Health Services Utilization (MHSU) was operationalized as a composite outcome (none, partial, adequate) based on ANC attendance, place of delivery, maternal and child PNC. Multilevel multinomial logistic regression models were fitted to assess individual, household, and contextual predictors. Variables significant at *p* < 0.20 in bivariate analysis were included in multivariable models. Statistical significance was set at *p* < 0.05.

**Results:**

Overall, 44.0% of women did not utilize any services, 44.4% partially utilized services, and only 11.6% achieved adequate utilization. Women with secondary or higher education were significantly more likely to achieve adequate utilization (Relative Risk Ratio (RRR) = 12.10, 95% CI: 8.72, 16.81). Wealth status, media exposure, employment, and health insurance were positively associated with utilization, while higher parity and decision-making barriers reduced utilization. Rural residence was associated with lower utilization (RRR = 0.38, 95% CI: 0.29, 0.49). Distance to health facilities was not significant in the adjusted model.

**Conclusion:**

Maternal healthcare service utilization in Nigeria remains suboptimal, with significant inequities driven by socioeconomic status, education, and women’s autonomy. Strengthening the continuum of care requires multisectoral interventions addressing structural and social determinants of health.

## Introduction

Maternal mortality remains a critical global public health challenge, with approximately 287,000 maternal deaths recorded worldwide in 2020, the vast majority of which occurred in low- and middle-income countries (LMICs) and were largely preventable [1,2]. Sub-Saharan Africa accounts for nearly 70% of the global burden, reflecting persistent inequities in access to quality maternal healthcare services [1,3]. Despite global commitments under the Sustainable Development Goals (SDGs), progress in reducing maternal mortality has been uneven, particularly in high-burden countries [3].

The continuum of maternal healthcare services utilization has been widely recognized as an effective strategy for improving maternal and neonatal outcomes. This approach emphasizes the integrated utilization of essential services across pregnancy, childbirth, and the postnatal period, including antenatal care (ANC), skilled delivery, and postnatal care (PNC) [4,5]. Completion of this continuum is critical, as discontinuities at any stage significantly increase the risk of adverse maternal and neonatal outcomes [5,6]. In line with this, the World Health Organization (WHO) recommends a minimum of eight ANC contacts during pregnancy to optimize health outcomes [7]. However, evidence from LMICs indicates that many women do not complete this continuum, with substantial drop-offs between service components [5,8].

Nigeria remains one of the countries most affected by poor maternal health outcomes, contributing disproportionately to the global burden. Recent estimates indicate that Nigeria accounts for over one-quarter (28.3%) of all maternal deaths worldwide, with approximately 8,200 deaths and a maternal mortality ratio of 1,047 per 100,000 live births [3]. Although a considerable proportion of women access at least one ANC visit, only 43% of births are assisted by skilled birth providers, while 39% of births occur in health facilities and 59% take place at home [9–11]. These discontinuities underscore systemic challenges within the healthcare system, as well as socioeconomic and cultural barriers influencing maternal healthcare utilization.

Nigerian women from poor households face financial barriers to accessing care, including direct healthcare costs and transportation expenses, while those living in rural and underserved communities experience greater difficulties due to long distances, poor road networks, limited transportation, and inadequate availability of skilled providers and health facilities [12–15]. In addition, women's autonomy in healthcare decision-making and the influence of husbands and other family members often shape decisions regarding when and where to seek maternal healthcare [12,16,17]. Sociocultural preferences for home delivery and the use of traditional birth attendants further contribute to lower utilization of skilled and facility-based maternity care [14,17,18].

Several studies have identified multiple determinants of maternal healthcare utilization in Nigeria and similar settings. Individual-level factors such as maternal education, parity, employment status, and media exposure, alongside household and contextual characteristics including wealth status, place of residence, and regional disparities, have been consistently associated with service uptake [19,20]. Additionally, women’s autonomy and decision-making capacity particularly regarding financial access play a crucial role in influencing healthcare utilization [19,21]. However, much of the existing evidence has focused on isolated components of maternal healthcare rather than examining the continuum of care as a composite outcome.

Therefore, this study aims to examine the determinants of continuum of maternal healthcare utilization in Nigeria using data from the 2024 Nigeria Demographic and Health Survey (NDHS). Specifically, it operationalizes maternal health service utilization as a composite outcome encompassing antenatal care attendance, place of delivery, and postnatal care for both mother and child, and applies a multilevel multinomial logistic regression framework to identify individual, household, and contextual factors associated with varying levels of utilization.

## Methods

### Study design and data source

This study is a secondary analysis of data from the 2024 Nigeria Demographic and Health Survey (NDHS), a nationally representative cross-sectional household survey conducted by the National Population Commission of Nigeria with technical support from the Demographic Health Survey (DHS) program and funding from the United States Agency for International Development (USAID). The NDHS employs a stratified two-stage cluster sampling design. In the first stage, enumeration areas are selected as primary sampling units, stratified by state and urban or rural residence. In the second stage, households within selected clusters are systematically sampled. All women aged 15–49 years who were usual residents of selected households or who slept in the household the night before the survey were eligible for interview. The analytic sample for this study comprised 13,596 women with a recent live birth (weighted n = 14,183) for whom complete data on all outcome components were available. Full details of the NDHS sampling procedures and questionnaires [22]. The NDHS received ethical approval from the National Health Research Ethics Committee of Nigeria. Secondary analysis of publicly available DHS data does not require additional institutional ethics approval.

### Variables

#### Outcome variable.

The primary outcome was Maternal Health Services Utilization (MHSU), operationalized as a composite indicator derived from four service components: (1) antenatal care attendance, classified as inadequate (fewer than eight visits) or adequate (eight or more visits), consistent with WHO recommendations; (2) place of delivery, classified as non-facility-based or facility-based; (3) postnatal care for the child, classified as received (yes) or not received (no); and (4) postnatal care for the mother, classified as received (yes) or not received (no). These four binary components were combined to create a three-category ordinal outcome: not utilized, defined as women who did not use any of the four service components; partially utilized, defined as women who used one, two, or three of the four components; and adequately utilized, defined as women who used all four components. The not-utilized category served as the reference group in all regression analyses.

#### Independent variables.

Individual-level covariates included: age group (15–24, 25–34, 35 or older), education level (none, primary, secondary or higher), current marital status (unmarried, married), current employment status (not employed, employed), birth interval (less than 24 months, 24 months or more, not applicable for first births), parity (continuous), media exposure (not exposed, exposed), decision-making autonomy (not a big problem, a problem), and medical insurance coverage (no, yes). Media exposure was defined as exposure to radio or television. Decision-making autonomy was operationalized as a binary variable indicating whether the respondent reported that obtaining permission to seek medical care or obtaining the money needed for treatment was a big problem; women reporting either barrier was classified as having a decision-making problem. Household and contextual variables included: wealth index (poor, middle, rich), place of residence (urban, rural), distance to health facility (not a big problem, big problem), and sex of household head (male, female).

### Statistical analysis

Descriptive statistics were computed for all study variables, and the distribution of MHSU was examined across all predictors. Weighted chi-squared tests for categorical variables and the Kruskal-Wallis test for parity were used to assess bivariate associations with MHSU. All estimates accounted for the complex survey design using sampling weights, clustering, and stratification.

Multilevel multinomial logistic regression was used to model the three-category MHSU outcome while accounting for the clustered structure of the DHS data, in which women are nested within enumeration areas. Before fitting the main models, a null model containing only the random intercept (Model 0) was estimated to assess the extent to which clustering at the enumeration area level contributed to variation in MHSU. The intra-class correlation coefficient (ICC) was computed for each outcome category relative to the reference category using the formula:


$$\begin{array}{c} ICC=\frac{\sigma_{u}^{\text{2}}}{\sigma_{u}^{\text{2}}+\frac{\pi^{\text{2}}}{\text{3}}} \end{array}$$

where π²/3 ≈ 3.29 represents the variance of the standard logistic distribution. An ICC greater than 0.10 was considered indicative of meaningful clustering, justifying the use of multilevel modelling [23]. The multilevel multinomial model is expressed as:


$$\begin{array}{c} n\left(\frac{P(Y_{ij}=k)}{P(Y_{ij}=B)}\right)=\beta_{\text{0}k}+X_{ik}^{\prime }\beta_{k}+u_{ik} \end{array}$$

where $P(Y_{ik}=k)$ is the probability of subject i in category k, $X_{ik}^{\prime }\beta_{k}$ are the fixed effects for predictors X on category k, $u_{ik}$ is the random effect for individual i in category k and $u_{i}\sim N\left(\text{0},\;\sigma_{u}^{\text{2}}\right).$

Multivariable analyses were conducted sequentially across three models. Model 1 included individual-level variables only; Model 2 included household and contextual variables only; and Model 3 included all variables simultaneously. Potential confounders identified based on theoretical considerations and existing literature were considered for inclusion regardless of their statistical significance in bivariate analyses. All variables with p < 0.20 in bivariate analysis were considered eligible for inclusion in the multivariable models. Model comparisons were done using Akaike Information Criteria (AIC) and Bayesian Information Criteria (BIC). Multicollinearity was assessed using the Variance Inflation Factor (VIF) and variables with VIF < 5 were included in the final model. Results are expressed as relative risk ratios (RRR) with 95% confidence intervals (CI). Statistical significance was set at p < 0.05 for all multivariable analyses. Missing data were handled using complete case analysis. All analyses were conducted in Stata 15.0, incorporating survey weights and the complex sampling design throughout.

## Results

### Sample characteristics

A total of 13,596 women of reproductive age were included in the analysis (weighted n = 14,183). In this sample, almost half were aged 25–34 years (49.0%), nearly half of the participants (47.1%) had no formal education, and 41.5% had attained secondary or higher education. Almost all respondents were married (95.6%), and more than half (56.2%) were currently employed. Media exposure was approximately equal, and the median parity was almost 3 (IQR: 2–5). Most women (87.5%) reported that decision-making was not a major problem, and most respondents (97.5%) had no medical insurance. At the household level, nearly half (47.8%) of respondents were classified as poor. The majority (62.5%) resided in rural areas, and only 27.7% reported that distance to a health facility was a significant problem. Most households were male headed (89.7%). Full sample characteristics are presented in Table 1.

**Table 1:** Individual and household/contextual characteristics of the sample (n = 13,596; weighted n = 14,183).

Characteristic	Category	Count (%)
***Individual***		
Age	15–24	3960 (27.9)
	25–34	6949 (49.0)
	≥ 35	3274 (23.1)
Education level	None	6682 (47.1)
	Primary	1615 (11.4)
	Secondary or higher	5886 (41.5)
Current marital status	Unmarried	623 (4.4)
	Married	13560 (95.6)
Currently employed	No	6209 (43.8)
	Yes	7974 (56.2)
Birth interval	< 24 months	2034 (14.3)
	≥ 24 months	9422 (66.4)
	NA	2727 (19.2)
Media exposure	Not exposed	7033 (49.6)
	Exposed	7150 (50.4)
Decision-making	Not a big problem	12405 (87.5)
	Big problem	1778 (12.5)
Medical insurance	No	13821 (97.5)
	Yes	362 (2.5)
Parity		Md = 3; IQR: 2–5
***Household and contextual***		
Wealth index	Poor	6775 (47.8)
	Middle	2851 (20.1)
	Rich	4557 (32.1)
Place of residence	Urban	5322 (37.5)
	Rural	8861 (62.5)
Distance to health facility	Not a big problem	10248 (72.3)
	Big problem	3935 (27.7)
Sex of household head	Male	12717 (89.7)
	Female	1466 (10.3)

**Note:** Md: Median, IQR: Interquartile range.

### Distribution of Maternal Health Services Utilization

Among the weighted overall sample of 14,183 women, 2,409 (17.0%) had adequate (eight or more ANC visits), 5,938 (41.9%) had facility-based deliveries, 6,158 (43.4%) received postnatal care for the child and for the mother, hence, 6,243 women (44.0%) had not utilized any maternal health service, 6,300 (44.4%) had partially utilized one to three services, and only 1,640 (11.6%) had adequately utilized all four services (Table 2). All individual and household/contextual characteristics were significantly associated with MHSU (p < 0.001).

**Table 2:** Distribution of maternal health service utilization by sample characteristics (weighted chi-squared results).

Characteristic	Not utilized (*n* = 6243)	Partially utilized (*n* = 6300)	Adequately utilized (*n* = 1640)	*p*
***Individual***				
**Age**				
15–24	1922 (30.8)	1776 (28.2)	262 (16.0)	<0.001
25–34	2914 (46.7)	3112 (49.4)	924 (56.3)	
≥35	1407 (22.5)	1412 (22.4)	454 (27.7)	
**Education level**				
None	4588 (73.5)	2017 (32.0)	77 (4.7)	<0.001
Primary	703 (11.3)	806 (12.8)	105 (6.4)	
Secondary or higher	952 (15.3)	3477 (55.2)	1457 (88.9)	
**Current marital status**				
Unmarried	204 (3.3)	333 (5.3)	87 (5.3)	<0.001
Married	6040 (96.7)	5967 (94.7)	1553 (94.7)	
**Currently employed**				
No	3454 (55.3)	2379 (37.8)	376 (22.9)	<0.001
Yes	2789 (44.7)	3921 (62.2)	1264 (77.1)	
**Birth interval**				
< 24 month	965 (15.5)	867 (13.8)	202 (12.3)	<0.001
≥ 24 month	4402 (70.5)	4036 (64.1)	984 (60.0)	
NA	876 (14.0)	1397 (22.2)	454 (27.7)	
**Media exposure**				
Not exposed	4387 (70.3)	2483 (39.4)	163 (9.9)	<0.001
Exposed	1856 (29.7)	3817 (60.6)	1477 (90.1)	
**Decision-making**				
Not a big problem	5161 (82.7)	5691 (90.3)	1553 (94.7)	<0.001
Big problem	1082 (17.3)	609 (9.7)	87 (5.3)	
**Medical insurance**				
No	6216 (99.6)	6095 (96.8)	1510 (92.1)	<0.001
Yes	26.7 (0.4)	205 (3.2)	130 (7.9)	
**Parity**	Md = 4; IQR: 2–6	Md = 3; IQR: 2–5	Md = 2; IQR: 1–4	<0.001
***Household and contextual***				
**Wealth index**				
Poor	4441 (71.1)	2236 (35.5)	98 (6.0)	<0.001
Middle	1221 (19.6)	1448 (23.0)	182 (11.1)	
Rich	581 (9.3)	2616 (41.5)	1360 (82.9)	
**Place of residence**				
Urban	1143 (18.3)	2903 (46.1)	1276 (77.8)	<0.001
Rural	5100 (81.7)	3396 (53.9)	364 (22.2)	
**Distance to health facility**				
Not a big problem	4162 (66.7)	4719 (74.9)	1367 (83.3)	<0.001
Big problem	2082 (33.3)	1580 (25.1)	273 (16.7)	
**Sex of household head**				
Male	5868 (94.0)	5509 (87.4)	1341 (81.8)	<0.001
Female	375 (6.0)	791 (12.6)	299 (18.2)	

Md: Median, IQR: Interquartile range, *p*: Chi-square *p*-value for categorical variables, Kruskal-Wallis test *p*-value for ‘Parity’.

Non-utilization was highest among women with no education (73.5%), no media exposure (70.3%), poor wealth status (71.1%), and rural residence (81.7%). Conversely, adequate utilization was highest among women with secondary or higher education (88.9%), media exposure (90.1%), rich wealth status (82.9%), and urban residence (77.8%). Median parity declined significantly across utilization categories, from 4 (IQR: 2–6) among non-utilizers to 3 (IQR: 2–5) among partial utilizers and median = 2 (IQR: 1–4) among adequate utilizers. Women from female-headed households were proportionally more represented in the adequately utilized group (18.2%) compared to the not utilized group (6.0%).

### Null model (Model 0)

Results from the null model (Table 3) indicate that the ICC values for the partially and adequately utilized outcome categories were significant (greater than 0.10), justifying the use of multilevel models.

**Table 3:** Null model results (Model 0).

	Partially versus not utilized		Adequately versus not utilized	
	RRR (95% CI)	*p*	RRR (95% CI)	*p*
Intercept	2.03 (1.83, 2.27)	<0.001	0.16 (0.13, 0.20)	<0.001

RRR: Relative risk ratio; CI: Confidence interval. ICC (partially vs. not utilized) = 0.43; ICC (adequately vs. not utilized) = 0.77. AIC = 21,882.32; BIC = 21,912.39. Reference category: not utilized.

### Individual-level predictors of MHSU (Model 1)

At the individual level, age, education, marital status, employment, media exposure, medical insurance, decision-making autonomy, and parity were significantly associated with MHSU, while birth interval (≥ 24 months) was not (p > 0.05) (Table 4).

**Table 4:** Individual-level multilevel multinomial logistic regression results (Model 1).

Characteristic	Partially versus not utilized		Adequately versus not utilized	
	RRR (95% CI)	*p*	RRR (95% CI)	*p*
**Age**				
15–24	Ref.		Ref.	
25–34	1.30 (1.14, 1.48)	<0.001	2.38 (1.90, 2.98)	<0.001
≥35	1.93 (1.60, 2.33)	<0.001	5.44 (4.02, 7.35)	<0.001
**Education**				
None	Ref.		Ref.	
Primary	2.22 (1.91, 2.57)	<0.001	7.27 (5.01, 10.54)	<0.001
Secondary or higher	5.09 (4.46, 5.82)	<0.001	31.71 (23.01, 43.69)	<0.001
**Married**				
Unmarried	Ref.		Ref.	
Married	1.11 (0.89, 1.38)	0.355	1.54 (1.10, 2.17)	0.013
**Employment**				
No	Ref.		Ref.	
Yes	1.46 (1.33, 1.62)	<0.001	2.04 (1.71, 2.44)	<0.001
**Birth interval**				
<24 months	Ref.		Ref.	
>=24 months	1.03 (0.90, 1.17)	0.677	1.03 (0.82, 1.29)	0.832
NA	1.60 (1.33, 1.92)	<0.001	1.88 (1.39, 2.53)	<0.001
**Media exposure**				
Not exposed	Ref.		Ref.	
Exposed	1.92 (1.72, 2.14)	<0.001	4.14 (3.34, 5.140	<0.001
**Decision-making**				
Not a big problem	Ref.		Ref.	
Big problem	0.67 (0.58, 0.77)	<0.001	0.51 (0.39, 0.68)	<0.001
**Medical insurance**				
No	Ref.		Ref.	
Yes	2.87 (1.65, 4.99)	<0.001	5.18 (2.85, 9.44)	<0.001
**Parity**	0.91 (0.89, 0.94)	<0.001	0.76 (0.72, 0.81)	<0.001

RRR: Relative risk ratio, CI: Confidence interval, p: p-value, ICC: Intraclass correlation coefficient, AIC: Akaike's information criterion, BIC: Bayesian information criterion. ICC1 = 0.21, ICC2 = 0.36, AIC = 19751.83, BIC = 19962.32.

Older age was positively associated with higher utilization; women aged ≥ 35 were over five times more likely to adequately utilize services compared to those aged 15–24 (RRR = 5.44, 95% CI: 4.02, 7.35). Education demonstrated the strongest association — the likelihood of adequate utilization of services in women with secondary or higher education was over 31 times that of none educated women (RRR = 31.71, 95% CI: 23.01, 43.69). Media exposure (RRR = 4.14, 95% CI: 3.34, 5.14), medical insurance (RRR = 5.18, 95% CI: 2.85, 9.44), and employment (RRR = 2.04, 95% CI: 1.71, 2.44) were similarly positively associated with adequate utilization. Married women were significantly more likely to adequately utilize services compared to unmarried women (RRR = 1.54, 95% CI: 1.10, 2.17), though marital status was not significant for partial utilization (p = 0.355).

Conversely, decision-making problems significantly reduced the likelihood of both partial (RRR = 0.67, 95% CI: 0.58, 0.77) and adequate utilization (RRR = 0.51, 95% CI: 0.39, 0.68). Each additional birth was associated with a reduced likelihood of adequate utilization (RRR = 0.76, 95% CI: 0.72, 0.81).

### Household and contextual predictors of MHSU (Model 2)

At the household and contextual level, wealth index, place of residence, and sex of household head were significantly associated with MHSU, while distance to health facility was not significant for either partial (RRR = 0.98, p = 0.753) or adequate utilization (RRR = 0.84, p = 0.100) (Table 5).

**Table 5:** Household and contextual-level multilevel multinomial logistic regression results (Model 2).

Characteristic	Partially versus not utilized		Adequately versus not utilized	
	RRR (95% CI)	*p*	RRR (95% CI)	*p*
**Wealth index**				
Low	Ref.		Ref.	
Middle	2.50 (2.18, 2.85)	<0.001	6.80 (4.98, 9.27)	<0.001
Rich	7.50 (6.35, 8.85)	<0.001	48.19 (35.00, 66.34)	<0.001
**Place of residence**				
Urban	Ref.		Ref.	
Rural	0.56 (0.47, 0.67)	<0.001	0.22 (0.16, 0.29)	<0.001
**Distance to health facility**				
Not a big problem	Ref.		Ref.	
Big problem	0.98 (0.88, 1.10)	0.753	0.84 (0.69, 1.03)	0.100
**Sex of household head**				
Male	Ref.		Ref.	
Female	1.53 (1.30, 1.79)	<0.001	1.81 (1.44, 2.27)	<0.001

RRR: Relative risk ratio, CI: Confidence interval, p: p-value, ICC: Intraclass correlation coefficient, AIC: Akaike's information criterion, BIC: Bayesian information criterion. ICC1 = 0.26, ICC2 = 0.46, AIC = 20369.73, BIC = 20474.97.

Wealth index demonstrated the strongest association in this model. Compared to low-wealth women, those in the rich wealth category were over 48 times more likely to adequately utilize services (RRR = 48.19, 95% CI: 35.00, 66.34), while middle-wealth women were nearly seven times more likely (RRR = 6.80, 95% CI: 4.98, 9.27). Rural residence was significantly associated with reduced likelihood of both partial (RRR = 0.56, 95% CI: 0.47, 0.67) and adequate utilization (RRR = 0.22, 95% CI: 0.16, 0.29) compared to urban residence. Women from female-headed households were significantly more likely to partially (RRR = 1.53, 95% CI: 1.30, 1.79) and adequately utilize services (RRR = 1.81, 95% CI: 1.44, 2.27) compared to those from male-headed households.

### Combined individual and household/contextual-level predictors of Maternal Health Services Utilization (Model 3)

In the combined model, all variables retained significance except birth interval (≥ 24 months) and distance to health facility (Table 6). Older age remained positively associated with utilization; women aged ≥ 35 were four times more likely to adequately utilize services compared to those aged 15, 24 (RRR = 4.00, 95% CI: 2.95, 5.43). Education remained the strongest individual-level predictor, where women with secondary or higher education were over 12 times more likely to adequately utilize services compared to those with no education (RRR = 12.10, 95% CI: 8.72, 16.81), though the magnitude attenuated compared to the individual-level model. Employment (RRR = 1.96, 95% CI: 1.64, 2.35), media exposure (RRR = 2.33, 95% CI: 1.86, 2.92), and medical insurance (RRR = 3.69, 95% CI: 2.03, 6.72) remained positively associated with adequate utilization. Married women were significantly more likely to adequately utilize services (RRR = 1.69, 95% CI: 1.18, 2.44), while marital status remained non-significant for partial utilization (p = 0.199). Decision-making problems significantly reduced the likelihood of both partial (RRR = 0.67, 95% CI: 0.58, 0.77) and adequate utilization (RRR = 0.54, 95% CI: 0.40, 0.73), and each additional birth remained inversely associated with adequate utilization (RRR = 0.80, 95% CI: 0.75, 0.85).

**Table 6:** Combined individual and household/contextual multilevel multinomial logistic regression results (Model 3).

Characteristic	Partially versus not utilized		Adequately versus not utilized	
	RRR (95% CI)	*p*	RRR (95% CI)	*p*
**Age**				
15–24	Ref.		Ref.	
25–34	1.20 (1.05, 1.37)	0.007	1.99 (1.58, 2.50)	<0.001
≥35	1.65 (1.37, 2.00)	<0.001	4.00 (2.95, 5.43)	<0.001
**Education**				
None	Ref.		Ref.	
Primary	1.92 (1.65, 2.23)	<0.001	4.25 (2.92, 6.19)	<0.001
Secondary or higher	3.46 (3.01, 3.98)	<0.001	12.10 (8.72, 16.81)	<0.001
**Married**				
Unmarried	Ref.		Ref.	
Married	1.16 (0.92, 1.47)	0.199	1.69 (1.18, 2.44)	0.004
**Employment**				
No	Ref.		Ref.	
Yes	1.43 (1.29, 1.58)	<0.001	1.96 (1.64, 2.35)	<0.001
**Birth interval**				
<24 months	Ref.		Ref.	
>=24 months	1.06 (0.92, 1.21)	0.420	1.08 (0.86, 1.360	0.515
NA	1.65 (1.37, 1.99)	<0.001	2.03 (1.50, 2.75)	<0.001
**Media exposure**				
Not exposed	Ref.		Ref.	
Exposed	1.49 (1.33, 1.67)	<0.001	2.33 (1.86, 2.92)	<0.001
**Decision-making**				
Not a big problem	Ref.		Ref.	
Big problem	0.67 (0.58, 0.77)	<0.001	0.54 (0.40, 0.73)	<0.001
**Medical insurance**				
No	Ref.		Ref.	
Yes	2.25 (1.30, 3.92)	0.004	3.69 (2.03, 6.72)	<0.001
Parity	0.93 (0.90, 0.96)	<0.001	0.80 (0.75, 0.85)	<0.001
**Wealth index**				
Low	Ref.		Ref.	
Middle	1.64 (1.43, 1.88)	<0.001	2.39 (1.75, 3.27)	<0.001
Rich	3.14 (2.63, 3.74)	<0.001	8.34 (6.03, 11.54)	<0.001
**Residence**				
Urban	Ref.		Ref.	
Rural	0.70 (0.59, 0.83)	<0.001	0.38 (0.29, 0.49)	<0.001
**Distance to health facility**				
Not a big problem	Ref.		Ref.	
Big problem	1.10 (0.98, 1.23)	0.105	1.02 (0.83, 1.26)	0.826
**Sex of household head**				
Male	Ref.		Ref.	
Female	1.33 (1.12, 1.57)	0.001	1.59 (1.26, 2.02)	<0.001

RRR: Relative risk ratio; CI: Confidence interval. ICC (partially vs. not utilized) = 0.19; ICC (adequately vs. not utilized) = 0.31. AIC = 19,270.77; BIC = 19,556.44. Reference category: not utilized.

At the household and contextual level, wealth index remained strongly associated with utilization; rich women were over eight times more likely to adequately utilize services compared to low-wealth women (RRR = 8.34, 95% CI: 6.03, 11.54), though attenuated from the household-level model. Rural residence remained inversely associated with both partial (RRR = 0.70, 95% CI: 0.59, 0.83) and adequate utilization (RRR = 0.38, 95% CI: 0.29, 0.49). Female-headed households remained positively associated with both partial (RRR = 1.33, 95% CI: 1.12, 1.57) and adequate utilization (RRR = 1.59, 95% CI: 1.26, 2.02). Distance to health facility was not significant in the combined model (p > 0.05).

## Discussion

This study examined the determinants of the continuum of maternal healthcare service utilization among women of reproductive age in Nigeria using nationally representative data from the 2024 NDHS. The findings reveal that the majority of women in Nigeria fail to complete the full continuum of maternal care: 44.0% did not utilize any of the four assessed service components, and only 11.6% achieved adequate utilization of all four. These patterns reflect a pervasive failure of continuity across the care pathway that is not unique to Nigeria but is characteristic of sub-Saharan Africa more broadly. Studies from Ethiopia and Tanzania have similarly documented low completion of the maternal care continuum, largely attributable to drop-offs between antenatal care and skilled delivery, and between delivery and postnatal care [24,25]. The Nigerian evidence presented here reinforces that improving maternal health outcomes will require strategies focused not only on expanding access to individual services but on sustaining women's engagement across the entire continuum of care.

Education emerged as the strongest individual-level determinant of maternal health service utilization in this study, a finding consistent with a large body of evidence from comparable settings. Women with secondary or higher education were over twelve times more likely to achieve adequate utilization in the fully adjusted model, and the magnitude of this association exceeded that of any other predictor. This is consistent with findings from Ghana, where higher educational attainment is strongly associated with increased antenatal care attendance and skilled birth attendance [26] and with evidence from Uganda and Kenya showing that education enhances women's capacity to recognize obstetric complications, navigate health systems, and demand quality care [27,28]. The attenuation of the education effect between Model 1 and Model 3, from an RRR of 31.71 to 12.10, is itself an important finding. It indicates that the strength of the association was reduced after accounting for household wealth, urban-rural residence, and other covariates included in the fully adjusted model. However, because this study used a cross-sectional association model and did not conduct formal mediation analysis, this attenuation should not be interpreted as evidence that household wealth or urban residence mediates the relationship between education and maternal health service utilization. The persistence of a strong, association between education and utilization after adjusting for these covariates underscores the importance of educational attainment as a key correlate of maternal health service utilization in Nigeria.

Household wealth demonstrated a strong and consistent association with maternal healthcare utilization across all models, though its effect attenuated substantially in the combined model, reflecting the confounding contribution of individual-level factors. In the household-only model, rich women were over 48 times more likely to achieve adequate utilization compared to poor women, a finding consistent with evidence from Uganda and Burkina Faso where financial constraints, including direct costs such as user fees and transport, as well as indirect costs such as time away from work and childcare, are identified as major barriers among women of lower economic status [29,30]. Even in the fully adjusted model, the wealth gradient remained large and statistically significant, indicating that financial access is an independent constraint on maternal care-seeking that is not fully explained by the higher educational attainment, urban residence, and media exposure that tend to accompany higher household wealth.

Rural residence was associated with lower utilization of maternal health services, consistent with findings across sub-Saharan Africa. In countries such as Malawi and Ethiopia, rural women report lower use of skilled delivery and postnatal care due to weaker health infrastructure and limited availability of skilled providers [31,32]. A notable finding in this study, however, is that self-reported distance to a health facility was not independently associated with utilization in either the household or the combined model after controlling for place of residence and other covariates. This pattern has been observed in other settings, including parts of Rwanda, where perceived quality of care, user costs, and sociocultural factors appear to exert greater influence on maternal care-seeking than physical distance alone [33]. The absence of a significant distance effect here may also reflect limitations in how DHS surveys capture access barriers: the binary categorization of distance as a problem does not capture variation in transport availability, road quality, travel time, or opportunity costs, all of which may be more proximate determinants of utilization than the simple self-reported measure. These considerations suggest that conventional survey measures may underestimate the true role of geographic access barriers and that their policy relevance should be interpreted with caution.

Media exposure and employment were both positively associated with maternal health service utilization. Studies from Ethiopia and Senegal indicate that exposure to mass media improves awareness of maternal health services and promotes care-seeking behavior [34,35]. Employment likely reflects financial independence and decision-making capacity, facilitating access to care. Decision-making autonomy itself was a critical determinant; women who reported difficulties in making health-related decisions were less likely to use services, consistent with evidence from Mali, where limited autonomy constrains timely maternal care [36]. Interventions promoting women’s empowerment and involving men in maternal health education could therefore enhance service utilization.

The inverse association between parity and adequate utilization is consistent with patterns reported in Ghana and Ethiopia, where higher-parity women are less likely to use formal maternal healthcare, possibly because prior uncomplicated deliveries reduce perceived need for professional care or because accumulated childcare burdens reduce the time and resources available for health service use [37,38]. This pattern is concerning because complications such as uterine rupture, postpartum hemorrhage, and obstructed labor may be more, not less, likely with increasing parity in settings with limited obstetric capacity. Targeted messaging that emphasizes the specific and persistent risks associated with each pregnancy, regardless of prior birth experience, is essential to address this misconception.

Women from female-headed households were more likely to utilize maternal health services, a finding that may reflect greater female control over household resources and health-related decisions in the absence of a male household head [39]. Although medical insurance coverage was very low in this sample (2.5%), it was significantly associated with higher utilization across all models. This is consistent with evidence from Ghana's National Health Insurance Scheme and Rwanda's community-based Mutuelles de Santé program, both of which have been shown to improve access to maternal care by reducing the financial barriers associated with direct out-of-pocket payments at the point of service [40,41]. The low coverage observed in this study reinforces the case for expanding insurance coverage in Nigeria, particularly through community-based and government-subsidized schemes that are accessible to rural and poor populations.

### Strengths and limitations

This study has several important strengths. The use of nationally representative data from the 2024 NDHS provides a large and geographically diverse sample that supports generalizable inferences about the determinants of maternal healthcare utilization across Nigeria's heterogeneous population. The multilevel analytical framework appropriately accounts for the clustered structure of the DHS data and allows the quantification of community-level contributions to variation in utilization over and above individual and household predictors, as confirmed by the ICC values from the null model. The composite operationalization of the continuum of care as a single three-category outcome represents an improvement over studies that examine individual service components in isolation and enables a more comprehensive assessment of continuity of care.

Several limitations must be acknowledged. The cross-sectional design of the NDHS prevents causal inference; the associations reported here should be interpreted as evidence of factors statistically associated with utilization rather than as evidence of causal effects. Self-reported ANC attendance is subject to recall bias and social desirability bias, particularly given the binary classification of adequate ANC as eight or more visits, a threshold that may be difficult to recall accurately over the typical reference period. The composite outcome itself may mask heterogeneity in service use patterns across the four components; future studies could examine the pathways through the continuum more granularly. The multilevel framework captures variation across enumeration areas but cannot account for unmeasured community-level factors such as health worker availability, facility quality, and sociocultural norms around childbirth, all of which may contribute to the observed cluster-level variation. Finally, the low prevalence of medical insurance in the sample (2.5%) limits statistical power for the insurance effect estimate, and results in this subgroup should be interpreted with caution.

## Conclusion

Maternal healthcare service utilization in Nigeria remains deeply suboptimal. Only one in nine women achieves adequate utilization of all four components of the maternal care continuum, and nearly half do not access any component at all. This study demonstrates that these patterns are not randomly distributed but are systematically shaped by education, household wealth, place of residence, women's decision-making autonomy, media exposure, employment, and health insurance coverage. The consistent and independent effects of these determinants across all three models confirm that inequities in maternal care utilization in Nigeria are embedded in multiple and interacting layers of social, economic, and structural disadvantage.

Addressing these inequities will require interventions that go well beyond conventional supply-side improvements in facility availability. Sustained investment in girls’ education is essential, not only as a direct determinant of maternal health service use but as a pathway to better economic outcomes, greater autonomy, and improved capacity to navigate health systems. Financial protection mechanisms, including expanded community-based and national health insurance coverage, are needed to reduce the cost barriers that prevent poor women from completing the continuum of care. Communication strategies through mass media and community health workers should be sustained and expanded, with particular attention to messages that address the specific risks of each pregnancy, regardless of prior birth experience and that engage men and families in supporting maternal care-seeking. Rural areas require targeted investment in infrastructure, skilled workforce deployment, and community health worker programs to reduce the utilization gap between urban and rural populations. Taken together, the evidence from this study points to the need for a comprehensive, multisectoral approach that simultaneously addresses structural barriers, strengthens women's empowerment, and ensures that financial and geographic access to the full continuum of maternal care is equitable across all segments of the Nigerian population.
